# Type 2 Diabetes: How Much of an Autoimmune Disease?

**DOI:** 10.3389/fendo.2019.00451

**Published:** 2019-07-04

**Authors:** Paola de Candia, Francesco Prattichizzo, Silvia Garavelli, Veronica De Rosa, Mario Galgani, Francesca Di Rella, Maria Immacolata Spagnuolo, Alessandra Colamatteo, Clorinda Fusco, Teresa Micillo, Sara Bruzzaniti, Antonio Ceriello, Annibale A. Puca, Giuseppe Matarese

**Affiliations:** ^1^IRCCS MultiMedica, Milan, Italy; ^2^Laboratorio di Immunologia, Istituto di Endocrinologia e Oncologia Sperimentale, Consiglio Nazionale Delle Ricerche (IEOS-CNR), Naples, Italy; ^3^Unità di NeuroImmunologia, Fondazione Santa Lucia, Rome, Italy; ^4^Dipartimento di Senologia, Oncologia Medica, IRCCS-Fondazione G. Pascale, Naples, Italy; ^5^Dipartimento di Scienze Mediche Traslazionali, Università Degli Studi di Napoli “Federico II”, Naples, Italy; ^6^Treg Cell Laboratory, Dipartimento di Medicina Molecolare e Biotecnologie Mediche, Università Degli Studi di Napoli “Federico II”, Naples, Italy; ^7^Dipartimento di Biologia, Università Degli Studi di Napoli “Federico II”, Naples, Italy; ^8^Institut D'Investigacions Biomèdiques August Pi i Sunyer (IDIBAPS), Barcelona, Spain; ^9^Centro de Investigación Biomédica en Red de Diabetes y Enfermedades Metabólicas Asociadas (CIBERDEM), Madrid, Spain; ^10^Dipartimento di Medicina e Chirurgia, Università di Salerno, Baronissi, Italy

**Keywords:** diabetes, autoimmunity, immunometabolism, inflammation, T cells

## Abstract

Type 2 diabetes (T2D) is characterized by a progressive status of chronic, low-grade inflammation (LGI) that accompanies the whole trajectory of the disease, from its inception to complication development. Accumulating evidence is disclosing a long list of possible “triggers” of inflammatory responses, many of which are promoted by unhealthy lifestyle choices and advanced age. Diabetic patients show an altered number and function of immune cells, of both innate and acquired immunity. Reactive autoantibodies against islet antigens can be detected in a subpopulation of patients, while emerging data are also suggesting an altered function of specific T lymphocyte populations, including T regulatory (Treg) cells. These observations led to the hypothesis that part of the inflammatory response mounting in T2D is attributable to an autoimmune phenomenon. Here, we review recent data supporting this framework, with a specific focus on both tissue resident and circulating Treg populations. We also propose that selective interception (or expansion) of T cell subsets could be an alternative avenue to dampen inappropriate inflammatory responses without compromising immune responses.

## Introduction

Type 2 diabetes (T2D) is a multifactorial and multilayer disease, characterized by an altered metabolism of glucose, fat and proteins. Hyperglycemia is the main common feature defining T2D, and clusters of patients are identifiable according to the specific combination of insulin resistance (IR) and absolute or relative insulin deficiency (1), a combination that leads to complex clinical trajectories underlying the development of early metabolic imbalances and later cardiovascular complications (2, 3). T2D and its complications remain major causes of morbidity and mortality in the western world (4).

While it is well established that type 1 diabetes (T1D) results from cell-mediated autoimmune pancreatic β cell destruction, T2D has been historically considered a metabolic disease, and metabolic determinants are traditionally identified as major pathogenetic factors. A more recent line of research has started to focus on low-grade inflammation (LGI) as a pervasive feature of T2D, accompanying the development and the progression of the disease, as well as the genesis of complications (5). Two of the main risk factors to develop T2D are aging and obesity, both known to promote tissue and systemic chronic inflammation, often referred to as inflammaging and metaflammation, respectively (5–8). A number of publications demonstrate that inflammation is not a mere bystander but it plays a key role in the progression of all the main features of T2D disease, e.g., IR, β cell failure or inability to cope with increased insulin demand, and atherosclerotic plaque development and destabilization (5–7). A plethora of putative inflammatory sources and mechanisms have been proposed to explain this evidence (5–9). Seminal discoveries and the majority of studies have mainly focused on cells of the innate immune system (10–12) and more recent data also suggest the direct involvement of acquired immunity. In particular, autoimmunity, a multifactorial process defined by loss of self-tolerance and chronic excess reactivity of B and T cells, has started to be recognized as an overlapping mark of both T1D and T2D. Furthermore, metabolic dysregulation and autoimmune components are able to generate a vicious cycle: the increased production of cytokines characterizing the chronic inflammatory state in T2D concur to destroy pancreatic β cells, and this inflammation-induced tissue damage leads to the release of “self” antigens that promote autoimmune activation. In turn autoimmunity further impairs insulin secretion in β cells and promotes hyperglycaemia (13–15).

A better recognition of the autoimmune components of T2D is relevant because it may lead to a deeper understanding of the mechanisms involved in the insurgence of hyperglycemia; it has also considerable therapeutic consequences, translating into a better classification and treatment of the disease. Indeed, T2D patients with a significant autoimmune component: (i) need insulin earlier during disease progression, (ii) are likely to poorly respond to classical anti-diabetic medications, (iii) may be highly responsive to immunomodulator therapy (16).

Our review focuses on existing evidence on autoimmune aspects of T2D from a molecular, cellular and clinical perspective. We describe how the knowledge regarding the intimate link between metabolism and inflammatory responses in specific immune cell populations is rapidly expanding, and report latest findings regarding the alteration of B and T (with a specific focus on T regulatory subsets) cells, the metabolic mechanisms driving their expansion or dysfunction in T2D (17–20). Finally, we briefly summarize promising clinical data regarding the potential of anti-inflammatory therapies in T2D and hypothesize a framework where inflammatory responses are modulated, rather than suppressed, to intercept and blunt the development and progression of the disease.

## Latent Autoimmune Diabetes of the Adult (LADA)

The presence of circulating autoantibodies in non-insulin-dependent diabetes mellitus was first identified more than 40 years ago (21). Nowadays, the presence of these autoantibodies characterizes a condition referred to as latent autoimmune diabetes of the adults (LADA). Autoantibodies against glutamic acid decarboxylase (GADA), islet cytoplasm (ICA), insulinoma-associated protein (IA-2A), and zinc transporter (ZnT8A) are commonly found in these patients.

Analyzing a large cohort of T2D patients (*n* = 3.672) from the UK, aged between 25 and 65 years, the percentage of subjects with ICA and/or GADA autoantibodies was found to be 12% overall and to be significantly higher in younger patients (22). Another study from the Pittsburgh cohort of the Cardiovascular Health Study found that also among diabetic patients aged over 65 years 12% had autoantibodies against GAD65 and/or IA-2, associated with an abnormal glucose control and a pronounced activation of the acute-phase response (increased fibrinogen and C-reactive protein levels), that may in part explain the observed defect in insulin secretion (23). A similar prevalence of diabetes autoimmunity was described in Argentinian elderly T2D patients (24). The largest European study (Action LADA) to date has later analyzed 6,156 T2D patients (age range, 30–70 years) for GADA, IA-2A and ZnT8A and found that 9.7% were positive, with the majority (8.8%) being GADA positive, and that, at diagnosis, these patients are usually non-insulin requiring and do not show categorically distinct clinical features from autoantibody-negative T2D patients (25). Ethnicity may count, as GADA positivity in T2D patients range from 3.8% in Japan (Eihme Study, *n* = 4,980) (26) to 10% in Norway (HUNT Study, *n* = 1,134) (27).

At diagnosis, LADA patients do not usually need exogenous insulin and they appear to be clinically affected by T2D, but a large percentage will need it within a few years, showing a much faster decline of β cell function compared to T2D patients, possibly caused by the ongoing immune-mediated β cell destruction. Notably, Turner et al. showed that 94% of patients with ICA and 84% of those with GADA required insulin therapy by 6 years, compared with 14% of those without the antibodies (22). A small study has directly correlated the presence of islet autoantibodies with significantly lower acute insulin response when compared to that of the autoantibody-negative group, but observed similar peripheral IR, providing compelling evidence that the profound impairment of insulin secretion is plausibly determined by the immune-mediated injury of pancreatic β cells (28).

## LADA at the Intersection of Type 1 and Type 2 Diabetes

Although formally classified as T1D for the typical presence of autoantibodies, LADA patients present several clinical features that are mixed between T1D and T2D pathologies. Low birthweight results to be a risk factor for LADA of the same strength as for T2D, suggesting LADA etiology includes factors related to T2D (29). Furthermore, LADA is associated with factors well known to promote T2D, such as overweight, physical inactivity, smoking, and sweetened beverage intake, suggesting LADA may in part be preventable through the same lifestyle modifications as T2D (30). In particular, the risk of LADA in relation to overweight/obesity was studied in two large population-based reports from a Swedish case-control study and the Norwegian HUNT Study, whose findings support the hypothesis that, even in the presence of autoimmunity, factors linked to IR, such as excessive weight, could promote LADA onset (31). Metabolomics of LADA, T1D and T2D patients failed to identify a unique metabolite profile for any of the diabetes types. Instead, the metabolome varied along a C-peptide-driven continuum from T1D to T2D, with LADA being an intermediate and patients metabolically closer to T1D showing a faster progression to insulin therapy than those closer to T2D (32). On the other hand, a Danish study analyzing a cohort of 4,374 adults with newly diagnosed diabetes demonstrated that fasting C-peptide and GADA status, but not age at onset, are able to define groups of diabetic patients with clinically relevant differences in glycaemic control and cardiometabolic risk, suggesting that the borders between T1D and LADA may be less discrete than believed (33). Parallel studies also demonstrated that LADA is associated with lower prevalence of microvascular complications, lower mortality, and lower risk of cardiovascular events, compared with T2D (34, 35). The risk of LADA is substantially increased with family history of T1D disease but also, albeit significantly less so, of T2D disease (36). A meta-analysis from 16 independent case-control studies (8,869 cases and 20,829 controls total), aimed at determining the association of T1D and T2D gene variants, demonstrated that some of these polymorphisms are associated with the risk of LADA, further supporting the idea of LADA as a combination of both T1D and T2D and emphasizing its heterogeneity (37). The first genome-wide association study of LADA revealed how the leading genetic signals were principally shared with T1D, although positive genetic correlations genome-wide were registered also with T2D. Authors identified a novel independent signal at the known T1D locus harboring the 6-Phosphofructo-2-Kinase/Fructose-2,6-Biphosphatase 3 (PFKFB3) gene (38). This gene encodes a regulator of glycolysis and insulin signaling and thus it had been previously reported as a plausible biological candidate in T2D diabetes (39). PFKFB3 also causes a reduction in T cell glucose consumption and survival, which in turn impairs the immune response in autoimmune conditions (40), calling for further studies to determine whether this genetic factor is truly a distinguishing feature between adult and childhood-onset autoimmune diabetes (38).

To further complicate the picture, while subjects classified as T2D patients are by definition autoantibody negative, autoantibodies may however also fluctuate. The Norwegian HUNT study unveiled that about 3% of subjects classified as T2D show a transient autoantibody positivity associated with earlier disease onset, a pre-diagnostic evidence of autoimmune activity in a sub-group of T2D patients (41).

**Figure 1:**
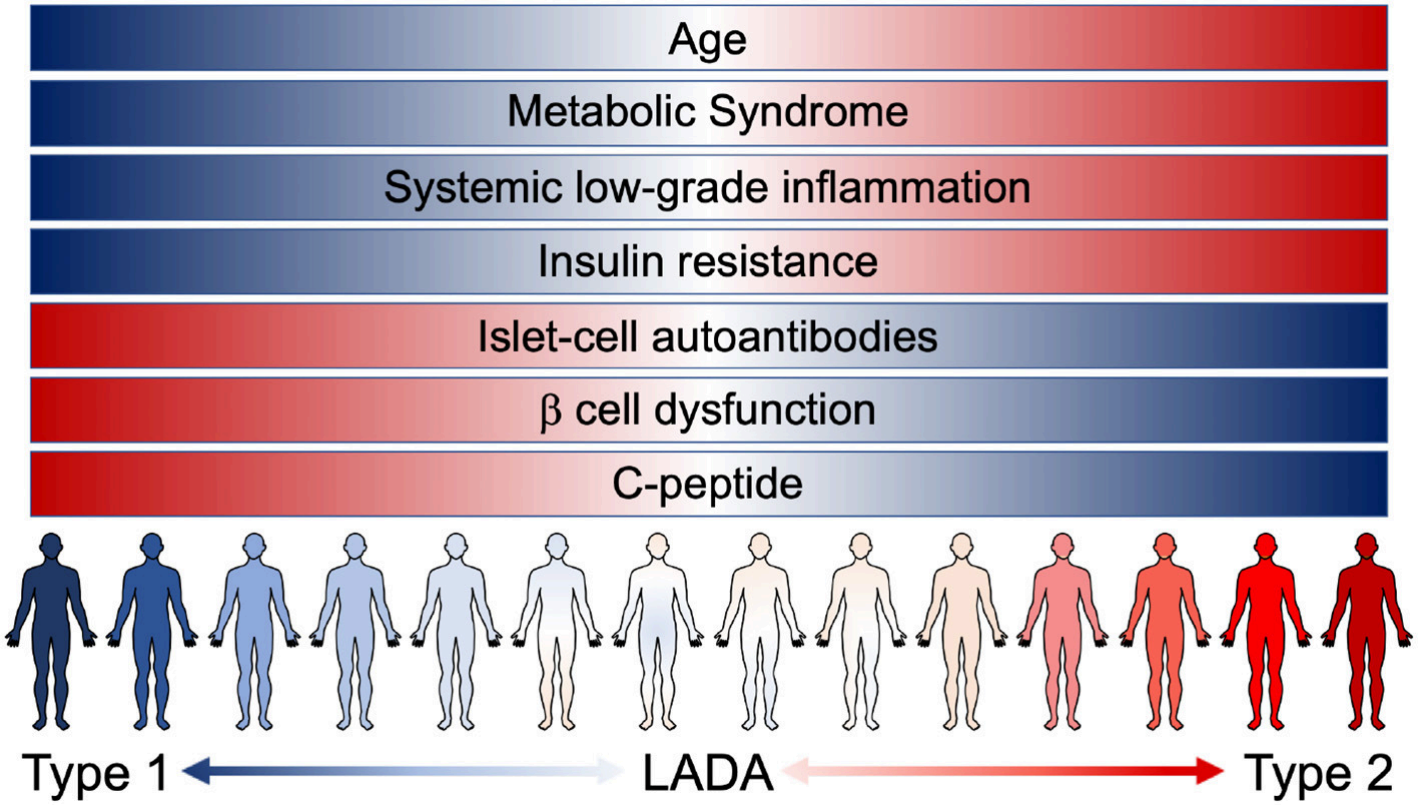
The continuous range of diabetes. Notwithstanding the different risk factors, instead of being clearly confined, T1D, LADA, and T2D patients are now known to present overlaying/overlapping clinical characteristics. LADA patients range from showing clear signs of β cell dysfunction (insulin deficiency and low levels of C-peptide) associated with strong markers of autoimmunity (presence of islet-cell autoantibodies) to patients showing a higher grade of insulin resistance and other pathological components resembling T2D condition [metabolic syndrome and systemic low-grade inflammation (LGI)]. The pathological features of the different forms of diabetes manifest as an uninterrupted spectrum that fails to clearly discriminate T1D and T2D.

LADA patients tend to share some clinical and phenotypic characteristics, compared to autoantibody-negative T2D patients: they are usually younger and leaner, suffer from acute symptoms and have a personal and/or familial history of autoimmune diseases (25). Nevertheless, LADA heterogeneity (from patients with clear signs of insulin deficiency associated with strong markers of autoimmunity, to patients showing weak markers of autoimmunity and closely resembling T2D) is possibly the mirror of a progressive and continuous clinical spectrum that blends, instead of discriminating, T1D and T2D (Figure 1). The crucial question arises: how important is the autoimmune component in autoantibody negative T2D patients? To try and answer to this question, we cannot abstain from describing the connection that exists between systemic metabolism and the immune system.

## The Immune-Metabolic Connection

Obesity, together with age, is the major risk factor for T2D. A large proportion of T2D patients are obese and the risk of T2D increases with increasing body mass index (BMI) (42). In both human and mice, obesity is associated with chronic LGI, thought to play a pivotal role in the development of IR and T2D pathological process (14). Although obesity is not yet a fully established risk factor for autoimmunity, the altered glucose tolerance and the development of IR due to an abnormal accumulation of adipose tissue (AT) in obese subjects coincide with an elevated incidence of autoimmune diseases (43). Epidemiological data analyzing T1D, multiple sclerosis, and psoriasis patients have recognized a causal risk factor for all these autoimmune conditions in an elevated BMI (44–46), although the mechanistic players of this association remain mostly undetermined. The coexistence of overweight and autoimmunity may be the result of concurrent dysregulation of immune tolerance, involving different organs (47). In the case of T2D, one of the most implicated districts is the AT (48).

The presence of enlarged fat depots that characterizes overweight/obese subjects, may be considered as an autoimmune pathology in which autoreactive T cells attack the adipocytes and fuel adipose inflammation (49, 50). On the other hand, AT is actually contiguous with main immune cell centers, such as lymph nodes, thymus, and bone marrow, in a close embrace that we have recently defined an “anatomical tango” (51). AT surrounds the thymus, hence may influence T cell differentiation in response to metabolic cues (52); furthermore, a high number of adipocytes resides in the bone marrow, with possible involvement in haematopoiesis, lymphopoiesis, and memory B and T cell responses (53). This physical contiguity has been noted by anatomists of the past, but only recently, though, it has been explored from a new and mechanistic point of view, and researchers have hypothesized and tested how it may allow a continuous communication (51).

The AT produces and releases several bioactive molecules, referred to as adipokines with a variety of pro-inflammatory and anti-inflammatory roles, which regulate both metabolism and immune cell function (20). Together with immune-cell derived cytokines, these immune metabolic mediators actually allow a reciprocal regulation between the AT and the immune system at both a paracrine and endocrine level (54, 55). One of the most biologically relevant of these molecules, leptin, is produced by the AT in proportion to the body fat mass and, at a systemic level, regulates appetite and food intake, promoting the entry of glucose into the tissues and reducing hyperglycemia (56). Leptin links lifestyle with inflammatory and/or autoimmune molecular mechanisms (57–59). It is not only known to significantly regulate the innate immune cells, but to also bridge obesity and dysregulated adaptive T cell activity. It seems that metabolic dysregulation both induces and depends on autoimmune alterations (60, 61). As a double edge sword, lymphocyte activation and function are intimately intertwined between metabolic disorders, including T2D, and inflammatory diseases (62, 63).

The research at the interface between the historically distinct disciplines of immunology and metabolism (immunometabolism) focuses on the regulatory connection between the metabolic state of an organism and immune cell physiology/activity. Unveiling this connection is endowed with enormous potential for T2D understanding (64).

While the involvement of AT-resident macrophages and innate immunity has been thoroughly analyzed (65, 66), fewer studies have focused on the role played by the adaptive immune B and T lymphocytes and their released factors in obesity-associated inflammation and T2D triggering events. Next, we critically review these studies.

## B Cell Alteration in T2D

B lymphocytes are responsible for the humoral immunity component of the adaptive immune system. Beyond antibody secretion, they can also present antigens and secrete cytokines. A plethora of B cell subsets have been identified according to the stage of differentiation, tissue localization and developmental lineage (67). Despite the controversies regarding the correspondence between human and murine B cells (68, 69), different alterations affecting both circulating and tissue-resident B cells have been described in T2D (70). Similarly, both antibody-mediated and contact-dependent mechanisms have been proposed in relation to B cell imbalance and loss of insulin sensitivity.

A clear demonstration supporting the role of B cells also in the development of IR has been provided in murine models. B cell–null New Zealand obese mice do not develop IR in response to obesity (71). Similarly, another study showed that either pharmacological or genetic depletion of B cells is sufficient to significantly attenuate immune cell infiltration and inflammation in the AT, finally promoting insulin sensitivity (72). Mechanistically, high fat diet (HFD) has been proposed to foster the early recruitment of B cells which promote T cell activation in the AT. In turn, this would boost M1 macrophage polarization, and thus induce IR. B cells can also exert detrimental effects systemically through the production of pathogenic IgG antibodies (73). Treatment of B cell-null mice with IgGs derived from WT mice exposed to HFD is sufficient to phenocopy the metabolic alterations induced by HFD (72). In addition, recent findings revealed that hyposialylated IgGs activate endothelial IgG receptor FcγRIIB to promote obesity-induced IR. Of note, the results were replicated with IgGs derived from patients with T2D and transferred to IgG-deficient mice, suggesting the relevance of this mechanism also for human diabetes (74). Consistently, human subjects with IR are characterized by 122 potential IgG targets (72). However, the wide range of possible antigens, their relatively low prevalence, and the absence of an evident tissue lesion (in any diabetes-relevant tissue) argue against a prototypical autoimmune phenomenon for T2D. On the other hand, a similar setting is compatible with low grade inflammation, a subclinical, chronic process without major manifestations of the acute inflammatory response (5). Similar findings expanded these results showing that B-T cell contact is mandatory to develop a pathogenic pro-inflammatory response. Indeed, the addition of a cytokine-permeable trans-well membrane blunted the B cell-induced Th17 response (75). Moreover, B cells from diabetic patients showed reduced secretion of the anti-inflammatory interleukin (IL)-10 upon stimulation (75), a phenomenon observed in multiple cell types exposed to the diabetic environment (9). Regarding specific B cell subpopulations, less is known about their deregulation in T2D, especially considering human data. Naïve B cells are divided into two subsets, the most abundant B-2 follicular and marginal zone B cells, responsible for generating the majority of high-affinity antibodies during an infection and the scarcer B-1 cells, arising from a different developmental pathway and able to generate natural antibodies in a T cell-independent manner (76). AT-resident, B-2 cells were identified to promote the inflammatory response to HFD and IR, possibly through a leukotriene B4 (LTB4) - LTB4 receptor 1 axis (77). Interestingly, two studies reported a protective role for both CD5^+^ B-1a and CD5^−^ B-1b cells [different at both phenotypic and development level (78)] in HFD-induced metabolic alterations in mouse models. Adoptive transfer of both B-1a and B-1b cells into HFD-fed, B cell–deficient mice ameliorated IR and glucose intolerance through IL-10 and polyclonal IgM-dependent mechanisms, while the transfer of B-2 cells worsened the metabolic imbalances (79, 80). Consistently, diabetic leptin receptor-mutant db/db mice had lower levels of peritoneal B-1a cells, which were also hypo-responsive in terms of differentiation to effector B cells and IgM production (81). On the other hand, one study reported conflicting results, since it showed that the suppression of B cell activation in AT of obese mice fail to discernibly affect systemic inflammation and glucose homeostasis (82). Findings in obese subjects demonstrate that B-1 cells and IgM antibodies in AT inversely correlate to inflammation and IR (80). Circulating populations of B cells in T2D patients further support a B-1 protective, B-2 detrimental paradigm since B-1a cell frequency are inversely correlated with HbA1c, LDL, and triglycerides, while B-2 cells show the opposite trend (83).

The resulting corollary to these observations is that patients with T2D may be characterized by an altered response to vaccination and a higher susceptibility to infections. B cell number and capacity to produce Ig are known to decline with age (84), while few data are available for T2D patients. Elderly patients are characterized by an altered response to vaccinations and infections, despite a higher basal pro-inflammatory status (84, 85). Similarly, B cells derived from T2D patients are characterized by a reduced ability to produce *de novo* antibody responses, despite a higher basal secretion of pro-inflammatory cytokines (86) but B cell repertoire appears to be affected by obesity, rather than by the diabetic status (87). Nonetheless, the incidence of a wide range of infective diseases is markedly increased in the diabetic population (88, 89). On the other hand, data regarding immune response to vaccination in T2D are not straightforward. In particular, unaltered or even optimal response to influenza vaccination has been reported in young and elderly patients with T2D (90–92). Guidelines for medical care strongly recommend annual influenza vaccination for diabetic patients (93) and a recent systematic review emphasizes and reinforces the need and value of seasonal vaccination to decrease severe complications, hospitalization and/or death in diabetic patients (94). Results regarding other vaccinations are sparser (95, 96).

Overall, more observational and mechanistic data are needed to identify the involvement of B cells in the development of IR and T2D. In particular, few prospective studies have been conducted in humans. Reported alterations in T2D patients could represent either a cause or a consequence of the disease. Nonetheless, the observations that (i) specific antibodies are associated with prevalent T2D (97); (ii) serum IgG2 levels are associated with whole-body insulin-mediated glucose disposal (98); and (iii) general abundance of circulating gamma globulins predicts incident T2D in a large cohort (99), support the postulate that B cell alteration plays a key role in T2D pathogenesis.

## CD4^+^CD25^high^ T Regulatory Cells, Masters of Tolerance

CD4^+^ T helper cells (in particular Th1 and Th17) are among the principal mediators of a pro-inflammatory environment, through the release of inflammatory cytokines (TNF-α, INF-γ, IL-17, IL-22, and IL-26 among others). CD4^+^CD25^high^Forkhead Box Protein P3 (FoxP3)^+^ T regulatory (Treg) cells, on the other hand, are a functionally distinct cell lineage committed to exert an anti-inflammatory/immune suppressive control on innate and adaptive immune responses and they represent the most relevant cells in the body to sustain immunological homeostasis (100, 101). Treg cells function by inhibiting the activity of the pro-inflammatory counterpart CD4^+^ Th1 and Th17 (also referred to as T conventional or Tconv) cell subsets (102, 103). Treg-mediated suppression is based on inhibitory molecules, anti-inflammatory cytokines such as IL-10, IL-35, and Transforming Growth Factor (TGF)β and Cytotoxic T-Lymphocyte Associated protein 4 (CTLA-4), metabolic modulation and direct cytolysis of target cells (101). The transcriptional factor FoxP3 is the master regulator of Treg cell phenotype and function. Treg cells can be subdivided according to their origin into two main groups: one directly originating from the thymus, and the other arising from the peripheral conversion of naive CD4^+^CD25^−^ Tconv cells (104). Peripheral Treg cell frequency ranges from 5 to 15% of CD4^+^ T cells, but the ratio between Treg and Tconv cells can diminish upon infection, in order to improve specific immune response activation (105). Notwithstanding the pivotal role of Treg cells in halting unwanted immune responses is certain (106), the contribution of their numerical and/or functional dysregulation to the very development and progression of human autoimmunity, and in particular autoimmune diabetes, is still to be completely clarified. The involvement and causal role of Treg cells in T2D pathogenesis is *a fortiori* still poorly defined. Relevant knowledge, though, has come from discovering that Treg cells are major sensors of the systemic metabolic state.

## Adipose Tissue-Resident Treg Cells in Mice

In 2009, the group of Diane Mathis showed that about 10% of stromovascular fraction from the visceral adipose tissue (VAT) of C57Bl/6 lean mice fall within the lymphocyte gate, close to half of which are of the CD3^+^ T lineage. Among them, they revealed the presence of a unique population of Treg cells, as a much higher fraction of the CD4^+^ T cell compartment than usually observed in lymphoid or other non-lymphoid tissues and, importantly, their numbers were strikingly and specifically reduced at this site in insulin-resistant models of obesity (107). VAT Treg cells show a phenotype clearly distinguishable from that of their counterparts in the spleen and lymph nodes, including a distinct gene-expression profile, T cell receptor repertoire, and pattern of chemokine and chemokine receptor expression. Cytokines differentially synthesized by VAT-resident Treg and Tconv cells directly affect the synthesis of inflammatory mediators and glucose uptake by cultured adipocytes. In particular, Treg cell elevated production of the anti-inflammatory cytokine IL-10 may be essential to curb AT inflammation (107). Moreover, the residence of Treg cells into AT expose them to high concentrations of the adipocytokines, *in primis* leptin, discovered to halt the generation and proliferation of these cells (60, 62, 108–110). Notably, Treg cells not only express the leptin receptor, but they also secrete leptin, directly contributing to metabolic homeostasis and glucose tolerance (60). Diane Mathis's group has also identified peroxisome proliferator-activated receptor (PPAR)-γ, the “master regulator” of adipocyte differentiation, as a crucial molecular orchestrator of VAT Treg cell accumulation, phenotype and function. By treating obese mice with pioglitazone, an antidiabetic agent whose mechanism of action resides in PPAR-γ activation, it was demonstrated that PPAR-γ expression in VAT Treg cells is necessary for drug-dependent restoration of insulin sensitivity (111). A more recent investigation showed that IL-33 signaling through its receptor ST2 and myeloid differentiation factor MyD88 are also essential for the development and maintenance of VAT-Treg cells and sustains their transcriptional signature. IL-33 administration actually induces vigorous population expansion of VAT Treg cells, which tightly correlates with improvements of metabolic parameters in obese mice (112). Gain-of-function experiments have provided more evidence on how VAT Treg cells are able to directly influence the inflammatory state of AT and, thus, IR in studies analyzing diet-induced obese, leptin-deficient ob/ob and db/db mice. Treg cells are able to decrease not only macrophage number, TNF-α and inflammation in VAT, but also pancreatic islet cell hyperplasia, liver fat accumulation, blood glucose, liver enzymes, IR and kidney damage (113, 114). Notably, restoration of Th1/Foxp3^+^ balance is able to reverse IR for months, despite continuing high-fat diet (115).

## Human Adipose Tissue-Resident Treg Cells

While the biological role of VAT-resident Treg cells seems fully established in mice, published data on human Treg cells have instead been conflicting.

FoxP3 mRNA is readily detectable in both omental and subcutaneous human fat depots and can be titrated by PCR. Despite missing access to non-obese controls due to the rarity of bariatric surgery on normal subjects, Feuerer et al. found a correlation between BMI and the drop in Treg cells in omental vs. subcutaneous fat, that suggested findings on mice may be translatable to humans (107). Another study further confirmed significant lower levels of FoxP3 gene expression in human omental samples in obese compared to lean controls (116). On the other hand, Zeyda et al. analyzed expression of marker genes specific for T cell subsets in visceral and subcutaneous AT from highly obese subjects and lean-to-overweight control subjects. Unexpectedly, proportions of cytotoxic T cells and Th1 cells were unchanged, whereas those of Treg cells were increased in VAT from obese compared to control subjects, and positively correlated with systemic and AT inflammation, thus failing to support a role of protective Treg cell loss in AT inflammation in obese subjects, as clearly indicated by mice studies (117). When FoxP3 mRNA level was quantified in obese subjects differing for insulin sensitivity, it was found decreased in obese insulin-sensitive, but not in insulin resistant patients as compared with lean control subjects. Actually, peripherally induced adaptive FoxP3 Treg cells seem to accumulate in the inflammatory adipose microenvironment in obese subjects with overt IR. Differently, Helios, mainly associated to thymus-derived Treg cells, was significantly lowered in human VAT of all obese subjects irrespective of their IR. Authors themselves commented on the incapability to exclude that FoxP3 mRNA expression may reflect other CD4^+^ T cells infiltrating VAT of obese subjects with IR (114). A very recent comprehensive investigation into how omental AT immunity changes with obesity and T2D in humans, still revealed important similarities but also differences to paradigms in mice. Patients with T2D had increased proportions of inflammatory cells, including M1 macrophages, with positive correlations to BMI. Treg frequencies did negatively correlate with BMI but were similar in T2D and non-T2D subjects (118). Compared to thymic population, omental AT Treg cells expressed higher levels of PPAR-γ, but failed to show any detectable expression of IL-33 receptor, ST2 (118), thus contradicting a previous report (112).

In 2014, Benoist and co-workers analyzed blood circulating Treg and Tconv cells from 168 donors, either healthy or with established T1D or T2D by performing genome-wide expression profiling. They described a Treg specific transcriptional signature, composed of some very variable transcripts and some almost invariant, with more extensive variability for genes that control effector function than for lineage-specification factors. This Treg signature became sharper with age and with increasing BMI, suggesting a tuning of Treg function with repertoire selection and/or chronic inflammation. Notably, none of the transcripts showed significant association to diabetes, and overall expression of the Treg signature was subtly perturbed in T1D, but not T2D, patients (119).

**Figure 2:**
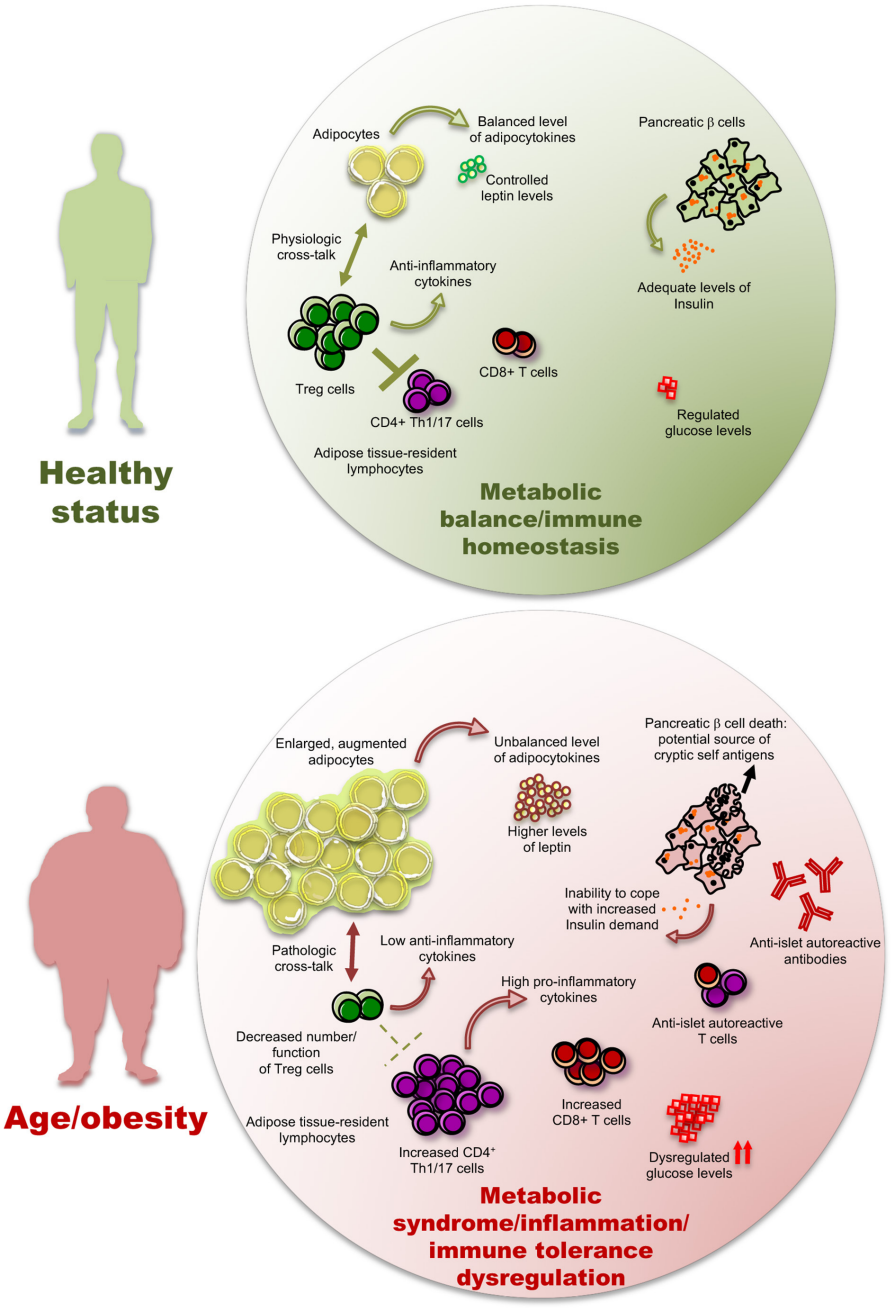
The inflammatory/autoimmune components of type 2 diabetes. Low-grade inflammation (LGI) induced by a high adipocyte mass, together with an elevated abundance of pro-inflammatory cytokines and adipocytokines produced by fat tissue, may hamper the number and function of anti-inflammatory Treg cells and diminish the level of anti-inflammatory cytokines, hence promoting immune responses by the CD4^+^ and CD8^+^ T cell subsets, including those with an autoreactive potential. The pancreatic β cell death leads to two pathological consequences in a vicious cycle of autoimmunity and metabolic dysregulation: (i) the potential increase of pancreatic cryptic self-antigens and (ii) a diminished ability to respond to insulin demand, with consequent further dysregulation of glucose levels. The presence of these phenomena is possibly significantly variable from patient to patient.

In Figure 2, we have schematized a possible involvement of adipose resident Treg cells in human T2D pathogenesis but studies on human AT-resident Treg cells are still too scant to provide with conclusive results. In particular, in the next future, it will be necessary to untangle whether in T2D patients Treg cells result further dysregulated, and how, compared to merely obese subjects.

## CD4^+^ T Conventional Cell Dysregulation in T2D

Treg cells are central in orchestrating the suppression of autoreactive responses by inhibiting the action of Th1 and Th17 cells counterpart. At the central core of autoimmune disease development, there is an imbalance between pro-inflammatory Th1/Th17 and anti-inflammatory Treg (120). Is this imbalance actually observed also in T2D?

In 2006, Seyfert-Margolis et al. published the development of a T cellular immunoblotting assay, with excellent sensitivity and specificity for measuring islet-specific T cell responses in autoimmune diabetes (121). In pivotal works of subsequent years, the same group used this approach to also screen adult phenotypic T2D patients, in parallel with detection of islet autoantibodies. Authors succeeded in identifying a group of adult autoimmune phenotypic T2D patients who are autoantibody-negative but T cell reactivity-positive, and showed that T cell responses to islet proteins fluctuate less than autoantibody responses and are associated with a more severe β cell damage and lower residual insulin secretion. It was concluded that these autoimmune patients would have not been recognized using autoantibody testing alone and thus islet autoimmunity may be more prevalent in adult phenotypic T2D patients than previously estimated (122–124). T cells from both T1D and T2D patients are capable to recognize islet proteins, but these proteins are not the same (123). The development of islet autoimmunity may perhaps follow different pathways in T1D compared to T2D, that is not unexpected since autoimmunity in T2D patients possibly arises as a sequela of obesity-dependent chronic inflammatory responses (125).

Beside self-reactive T cells, obesity and T2D are associated with an imbalance in T cell sub-population ratio. In mice, diet-induced obesity selectively promotes an expansion of the Th17 T cell lineage, a subset with a prominent pro-inflammatory role, and progressively higher production of IL-17 than lean littermates. A pronounced autoimmunity, meaning more severe early disease and histopathology, in two disease murine models of multiple sclerosis and inflammatory bowel disease is known to associate with an increased IL-17 producing Th17 cell bias in target tissues in target tissues (central nervous system and colon, respectively) (126). Jagannathan-Bogdan et al. found that *ex vivo* T cells from T2D patients appear to be poised for IL-17 production and have increased production of IFN-γ. In contrast, T2D patients had decreased percentages of Treg cells, suggesting that T cells in T2D patients are naturally skewed toward pro-inflammatory subsets that likely promote chronic inflammation in T2D through elevated cytokine production (127). Another study demonstrated that the ratios of Th17/Treg cells and Th1/Treg cells were significantly increased in T2D patients. In particular, the peripheral induced Treg cell number was reduced and Treg cells seemed more prone to cell death, since they showed a decreased Bcl-2/Bax ratio (128).

When TNF receptor superfamily member 5 (CD40)/CD40 ligand (CD40L) pathway, central player in the onset and maintenance of the inflammatory reaction, was studied in human AT, a direct cross-talk between adipocytes (CD40-expressing) and lymphocytes (CD40L expressing) was described, based on actual cell-cell contact (129). CD40 mRNA levels were significantly higher in subcutaneous than in visceral AT of obese subjects and were positively correlated with BMI, and with IL-6 and leptin mRNA levels. T lymphocytes were thus hypothesized to regulate adipocytokine production through both the release of soluble factor(s) and heterotypic contact with adipocytes involving CD40, that may play a direct role in obesity-associated human AT inflammation (129).

What about the pancreas? It is conceivable that a systemic inflammatory state may expose the pancreatic insulin producing cells to immune-mediated damage by random and not specific pathways. LGI induced by a high adipocyte mass, together with an elevated abundance of pro-inflammatory cytokines and adipocytokines produced by fat tissue, promote immune responses by the Th1 and Th17 cell subsets, that favors generic bystander activation of pro-inflammatory immune cells, including those with an autoreactive potential (130). In other words: (i) stressed adipocytes secrete pro-inflammatory adipokines and cytokines, able to recruit macrophages but also B and T cells (131); (ii) pro-inflammatory macrophages and activated lymphocytes secrete cytokines and chemokines, that turn the inflammatory reactions persistent (132). These phenomena may favor the conditions for incidental development of β cell autoreactivity and promote the engagement and activation of self-reactive T cells with direct cytotoxic effects.

*In vitro* treatment of human pancreatic islets from nondiabetic organ donors with high glucose levels is able to induce Fas expression, caspase-8 and−3 activation, and β cell apoptosis (133). Importantly, an up-regulation of Fas in β cells of T2D patients relative to nondiabetic control subjects has been observed, suggesting elevated glucose levels may contribute to islet destruction by a mechanism based on the interception of β cell expressed Fas Receptor by Fas Ligand (constitutively expressed by many immune cells, including activated T cells), independently of a classic antigen-based autoimmune reaction (133). A subsequent work helped to substantiate that in this detrimental regulatory loop, Fas upregulation, DNA fragmentation, and impaired β cell function were preceded by increased production and release of IL-1β, followed by NF-κB activation in β cells, implicating an inflammatory process in the pathogenesis of T2D glucotoxicity (134). Using high-density microarray analysis of the β cell transcriptome, it was more recently discovered that of those potentially able to activate STAT-1 or NF-κB pathways, TNFR-5 is the most sensitive to high glucose and fatty acid environment, confirming the role of inflammatory pathways in triggering glucolipotoxic islet cell death (135). Experiments performed at the beginning of the 1990s revealed that high glucose is able to also increase β cell immunogenicity (136, 137). Exposing Osborne-Mendel rats to a high-sucrose and high-fat diet with insulin releasing effects was shown to increase both β cell function and islet antigen expression (136). In isolated Sprague-Dawley rat islets cultured at different glucose concentrations, GAD expression was found to be glucose dependent and to correlate with increased functional state of the β cell (137).

It is also important to consider that β cell death and destruction lead to a relevant consequence on the perspective of “self” recognition: the release of a potentially notable amount of “cryptic” β cell antigens with immunogenic potential (Figure 2). These cell-free antigens may now encounter self-reactive T cells, leading to further immune-mediated β cell death and islet destruction (138).

## Clinical Aspects of Autoimmunity in T2D

Since accumulating evidence show an etiopathological role of LGI in the development of T2D, clinical trials and observational studies have been designed to explore the effect of anti-inflammatory drugs on glucose parameters (139, 140). On the other side, also glucose-lowering drugs can have anti-inflammatory effects. The description of the pleiotropic, beneficial, anti-inflammatory effects of selected anti-diabetic medications is beyond the scope of this manuscript and it has been largely reviewed elsewhere (5, 6, 139, 141–143).

Given the role of IL-1β in both IR development and β cells deterioration, a number of trials tested different biological drugs blocking IL-1β pathway, e.g., anakinra and canakinumab. These agents have shown a significant beneficial effect on HbA1c, IR, and β cell secretory function (144–146). Some of the outcomes were also long-lasting, since the effect on proinsulin-to-insulin (PI/I) was visible 39 weeks after cessation of the intervention (146). However, the effect on HbA1c was neither enduring nor clinically relevant, if compared to those obtained with conventional glucose-lowering drugs. In addition, a long-term treatment with an immune suppressive agent could be accompanied by relevant adverse effects, especially considering that a continuous presence of the drug would be needed. On the other side, an LGI-targeting intervention could be beneficial in T2D even beyond glycemic control (6, 147). Indeed, a recent trial demonstrated a significant reduction of cardiovascular events in patients treated with canakinumab, forty percent of which constituted by T2D patients, already on optimal pharmaceutical polytherapy (148). Secondary analysis did not reveal a different trend in this specific population. On the contrary, the beneficial effect was particularly evident in patients with high C reactive protein at the beginning of the treatment and diminished levels at the end of the trial, suggesting the existence of a residual (despite polytherapy) inflammatory risk also for T2D (149).

The first discovery linking LGI and T2D found an increased expression of TNFα in the AT of obese subjects (10). A number of observational studies and small-pilot trials have tested the effect of anti-TNFα biologicals on glucose parameters, showing improved insulin sensitivity in subjects without diabetes and with chronic inflammatory diseases, such as rheumatoid arthritis (150–153). While the use of TNF-α antagonism is associated with an improved endothelial function in a wide range of patients (154, 155), however, it has not demonstrated a tangible benefit in patients with T2D (156, 157).

Salicylates are another class of anti-inflammatory drugs tested in settings of T2D. Low-dose aspirin is known to inhibit cyclooxygenase (COX) 1 and 2, while higher doses can also inhibit NF-kB (140). The first clinical trial showed that high-dose aspirin improved IR and both fasting and post-prandial hyperglycemia in patients with T2D (158). Later, the TINSAL-T2D study tested salsalate, a salicylate prodrug marginally affecting the platelet aggregation pathways, in a multicenter randomized trial, demonstrating a significant reduction of HbA1c (159). However, both aspirine and salsalate are associated with a plethora of adverse events, e.g., risk of bleeding, gastrointestinal and renal toxicity, which hampered their full translation in clinical settings (140). Of note, differently from the general population, aspirin use in T2D patients does not appear to provide CV protection in settings of primary prevention (160).

Other conventional immune suppressing drugs such as diacerein, chloroquine, and TLR inhibitors have been shown to affect some markers of glucose metabolism (139, 140). However, immune suppression is always accompanied by side effects, mainly associated with an increased risk of infective diseases (161, 162). In addition, the chronic nature of LGI in T2D implies that such treatments should be applied continuously to obtain enduring effects. Also, a putative beneficial, transient effect of selected inflammatory molecules on glucose homeostasis has been proposed (163). Two alternative approaches to target LGI without suppressing immune function may be represented by: (i) the removal of the pro-inflammatory triggers; (ii) the selective modulation of immune cells function. The first approach is already being exploited with specific interventions known to target LGI and to improve diabetes outcomes, e.g., diet, exercise, and bariatric surgery (5, 164). Also, innovative molecules aimed at attenuating fundamental triggers of LGI are going to be tested in T2D, e.g., microbiota modulators, epigenetic-modifying, and senescence-targeting drugs (5, 143). On the other hand, increasing evidence suggests that immune response can be selectively modulated, rather than suppressed. Indeed, systemic delivery of nanoparticles coated with autoimmune-disease-relevant peptides bound to major histocompatibility complex class II (pMHCII) molecules triggers the generation and expansion of antigen-specific regulatory CD4^+^ T cell type 1 (TR1)-like cells, restoring normoglycemia in spontaneously diabetic mice. Of note, the effect was evident independently of the prevalence of the disease-relevant antigen and without compromising systemic immunity (18). These promising results may be considered within the panorama of the continuously emerging role of extracellular vesicles (EV)s for the regulation of immune cell behavior. Indeed, EVs exert complex immunomodulatory effects on target cells, acting as both antigen-presenting modules and as shuttles for regulatory biological information (19). EVs can deliver molecules that exert opposite functions, serving as antigens of innate immune receptors or promoting pathogen immune evasion (165). Qualitative and quantitative alterations regarding EVs in settings of T2D are constantly emerging (166–168), as well as their central role in the regulation of immune function (20, 169, 170). Finally, a deeper understanding of this newly described extracellular vesicle-based cell-cell communication machinery will probably enable the design of specific nanodrugs able to produce a long-lasting beneficial effect on the pathological inflammatory component of T2D.

## Conclusions

Autoreactivity ranges from a low and physiological level that is actually necessary for proper lymphocyte selection and immune system homeostasis, to an intermediate level that displays circulating antibody with no major tissue infiltrates, to frank pathogenic autoimmunity with manifested immune-mediated tissue damage (47).

The current classification of T1D, T2D, and LADA presents challenges to the diagnosis and treatment of diabetic patients, due to conflicting and confounding definitions, that still fail to incorporate advances in our understanding of these diseases. In 2001, TJ Wilkin formulated a hypothesis to respond to the increasingly fuzzy distinctions between T1 and T2 diabetes mellitus both clinically and aetiologically. His “accelerator hypothesis” identifies three processes which variably accelerate β cell loss: constitution, IR, and autoimmunity. Rather than overlap between two types of diabetes, the accelerator hypothesis envisages overlay: body mass is central to the development and rising incidence of all diabetes. Only tempo distinguishes the “types” (171). A clinical study, led by TJ Wilkin and aimed at testing this hypothesis was launched in Scotland in 2016 by the name of Accelerator Prevention Trial (adAPT). The main objective of the trial is to evaluate the impact of metformin on β cell stress, immune response and incidence of T1D in otherwise healthy children at high risk of T1D. The clinical outcome (incidence of diabetes) is expected in 2023 (172).

Accumulating evidence suggests that B and T cell alterations precede the loss of insulin sensitivity in adipose tissue and contribute to the general pro-inflammatory drift observed in T2D (73). Over-nutrition and advancing age are held to foster an intricate immune trafficking in different diabetes-relevant organs, including liver, muscle, and the adipose tissue (5, 73, 173, 174). Lacking wide-spread identification of specific autoreactive T cell populations and the wide array of antibodies associating to IR do not support the view of a prototypical autoimmune phenomenon for T2D. However, some features of the inflammatory trajectory accompanying all the stages of T2D are likely ascribable to low-grade, subclinical, autoimmune phenomena (68, 69, 72, 115).

## Future Prospects

Despite the increasing knowledge regarding the inflammatory component of T2D, the existing relevant gap of information has hampered the development of effective anti-inflammatory treatment to manage, prevent, or even revert T2D. Indeed, all the clinical approaches tested so far have tried to suppress the inflammatory response by antagonizing a candidate cytokine or pathway with a putative prominent role, rather than modulating specific cell types and responses. Recent literature is now adding new pieces to the complex crosstalk between cells of innate and acquired immunity, such as extracellular vesicles. Coupled with a continuously progressing technology, these studies may eventually lead to the design of innovative nanoscale drugs able to intercept or expand specific population with pro- or anti-inflammatory characteristics, respectively.

The relevance of the autoimmune phenomena possibly underpinning T2D could be masked by the heterogeneity of the disease. Indeed, many clinicians invoke the case for more personalized data analysis with the aim to move toward precision medicine, that would allow targeting the specific pathological mechanisms of hyperglycemia of the single patient (175). A recently proposed new sub-stratification of diabetes identified five replicable clusters of patients based on six variables (glutamate decarboxylase antibodies, age at diagnosis, BMI, HbA1c, β-cell function, and IR). A similar approach has been proposed to select candidate patients for anti-inflammatory treatments in T2D (140). These efforts are aimed at individualizing treatment regimens and identifying subjects with increased risk of complications at diagnosis, and thus represents a first step toward precision medicine (176).

It remains worth keeping to pursue a better definition of autoimmunity in T2D because a deeper knowledge of its immunogenic basis is not only necessary to better discriminate patient characteristics and select proper treatment approach, but, also importantly, to fully understand the very pathological mechanism of disease, thus possibly developing new strategies to counteract T2D development and progression.

## Data Availability

No datasets were generated or analyzed for this study.

## Author Contributions

PdC, FP, and GM conceived the work and supervised the writing. SG, VD, MG, FD, MS, AlC, AnC, and AP wrote the different paragraphs. CF, TM, and SB conceived the art work.

### Conflict of Interest Statement

The authors declare that the research was conducted in the absence of any commercial or financial relationships that could be construed as a potential conflict of interest.
